# A rare case of transient left ventricular apical ballooning syndrome following living donor liver transplantation: A case report and literature review

**DOI:** 10.1016/j.ijscr.2019.02.002

**Published:** 2019-02-10

**Authors:** Asuka Tanaka, Takashi Onoe, Kohei Ishiyama, Kentaro Ide, Hirotaka Tashiro, Hideki Ohdan

**Affiliations:** a Department of Gastroenterological and Transplant Surgery, Applied Life Sciences, Institute of Biomedical and Health Sciences, Hiroshima University, Hiroshima, Japan; b Institute for Clinical Research, National Hospital Organization, Kure Medical Center/Chugoku Cancer Center, Kure, Japan

**Keywords:** CK-MB, creatine kinase MB, CTR, cardiothoracic ratio, ECG, electrocardiogram, EF, ejection fraction, hANP, human atrial natriuretic peptide, HCC, hepatocellular carcinoma, IABP, intra-aortic balloon pumping, ICU, Intensive Care Unit, LC, liver cirrhosis, LDLT, living donor liver transplantation, LV-ABS, left ventricular apical ballooning syndrome, MELD, Model for end-stage liver disease, NASH, nonalcoholic steatohepatitis, NT-pro BNP, N-terminal pro-brain natriuretic peptide, PBC, primary biliary cirrhosis, POD, post-operative day, PSC, primary sclerosing cholangitis, RFA, radiofrequency ablation, Cardiac complication, Living donor liver transplantation, Transient left ventricular apical ballooning syndrome, Case report

## Abstract

**Introduction:**

Transient Left Ventricular Apical Balloon Syndrome (LV-ABS) is an unknown acute dysfunction of the left ventricle, which develops under emotional or physical stress events like the major surgical postoperative period.

**Presentation of case:**

A 68-year-old woman diagnosed with hepatitis C cirrhosis was admitted for living donor liver transplantation (LDLT). No evidence for underlying cardiac or pulmonary disease was found. LDLT was performed, and her intra and post-operative course was uneventful. On post-operative day 4, dyspnea and tachycardia were observed. Echocardiography showed a severe wall motion abnormality with ballooning of the apex with normal basilar wall motion and a severely impaired left ventricular function with an ejection fraction (EF) of 40%. Blood tests showed greatly increased level of N-terminal pro-brain natriuretic peptide (NT- pro BNP). Evidence of significant ischemic heart disease was not obtained and a transient LV-ABS was diagnosed. Although she required intensive care, her cardiac function became normal by conservative treatment.

**Discussion:**

Transient LV-ABS has been reported to occur more frequently in post-menopausal women with reduced estrogens reported to protect myocardium from sympathetic stress after mental or physical stress events. It has been suggested that this disease arises from myocardial stunning with high levels of circulating catecholamines and stress related neuropeptides. However, even in a serious condition, cardiac function has recovered normally in all cases by appropriate care.

**Conclusion:**

Transient LV-ABS is one cause of cardiac dysfunction after LDLT, and it is important to strictly manage cardiac function when diagnosed as transient LV-ABS.

## Highlights

• Transient Left Ventricular Apical Balloon Syndrome (LV-ABS) is an acute dysfunction of the left ventricle.
• Its clinical feature is similar to myocardial infarction.
• The echocardiogram show characteristic finding which is different from that of myocardial infarction.
• LV-ABS is rare, however, should be considered after liver transplantation.
• Proper diagnosis is important to restore cardiac function although it improve almost normally with conservative treatment.

## 1 Introduction

Transient left ventricular apical ballooning syndrome (LV-ABS) is unknown acute dysfunction of the left ventricle, which develops under emotionally or physically stressful event such as the perioperative setting after minor or major surgical procedures including deceased donor liver transplantation [[1], [2], [3]]. The clinical feature mimics an acute myocardial infarction, although it shows unique echocardiographic features [[4], [5], [6]]. Characteristically, hypokinesis or akinesis occurs in the middle and apical segments of left ventricle in the absence of epicardial coronary lesions, which results in ballooning of the apical wall with sparing of basal systolic function. Prognosis of this disease is relatively good and cardiac function of most cases improves to normal levels with a proper conservative management [4,5]. Here, we report the case of transient LV-ABS in liver transplantation recipient several days after LDLT with some literature view. Although the patient temporarily required intensive care, the cardiac function recovered to normal level after conservative treatment. Our work has been reported in line with the SCARE criteria [7].

## 2 Presentation of case

A 68-year-old female patient had been diagnosed with hepatitis C cirrhosis along with hepatocellular carcinoma (HCC) and HCC was treated with radiofrequency ablation (RFA) therapy. After RFA therapy, her liver function deteriorated and she developed hepatic encephalopathy frequently. She was referred to our hospital as a candidate for living donor liver transplantation (LDLT). A preoperative Model for End-stage Liver Disease (MELD) score was 14 and there was no evidence of recurrent HCC.

Her past medical history was unremarkable, and there was no evidence for underlying cardiac or pulmonary disease. Preoperative echocardiography demonstrated normal left ventricular function (EF 65%) with normal left ventricular size and motion and a 12-lead electrocardiogram (ECG) showed no abnormal findings including ischemic change.

She underwent LDLT using left lobe graft from her son. Total operating time was 13.0 h and the amount of bleeding was 4700 ml. Despite a high volume transfusion (packed red blood cells 14 units, fresh frozen plasma 5 units; platelet cells 10 units) during transplantation, the intraoperative course was uneventful. The patient required vasopressors during the procedure, but had no cardiac event or hemodynamic instability. After the operation, epinephrine was administered to the patient (8.62 γ).

**Fig. 1:**
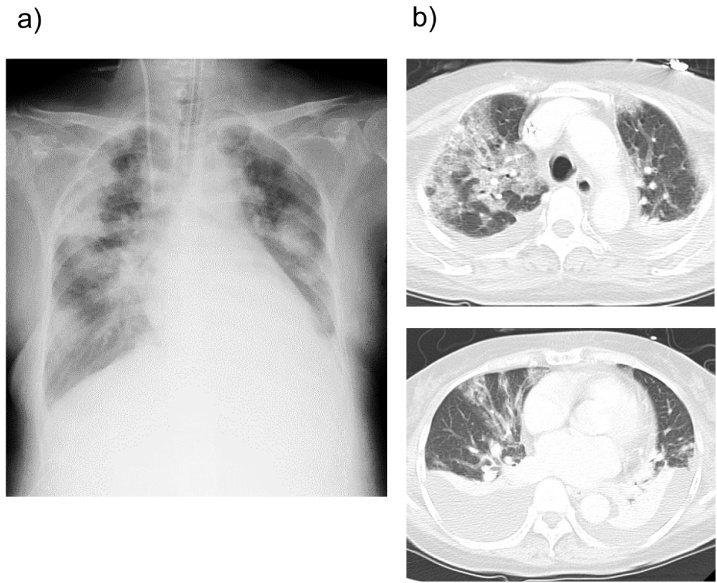
a) Radiograph showing an expanded cardiothoracic ratio (CTR) and bilateral field opacities in a butterfly distribution on POD 4. b) Chest CT showing diffuse ground-glass appearance and pleural effusion in bilateral lung fields.

**Fig. 2:**
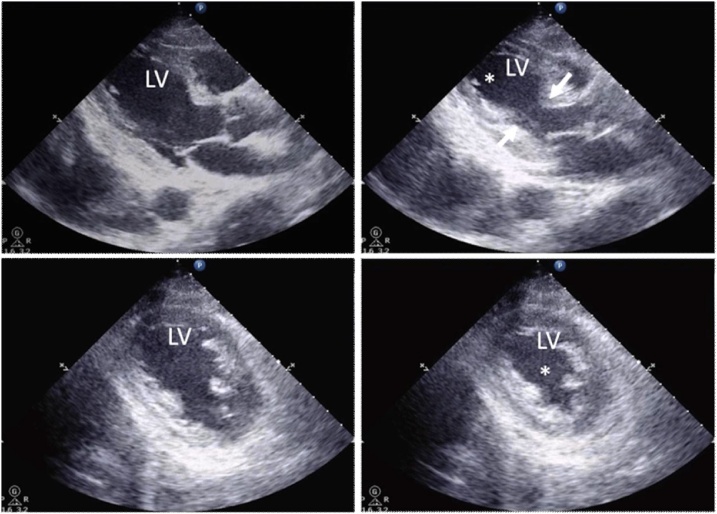
Echocardiogram on POD 4. Parasternal long axis views (top) and short axis views (bottom) of end-systolic frames (right) and end-diastolic frames (left) are shown. While the basal septum shows normokinesia (arrows), the apical septum is akinetic (*). LV; left ventricular.

**Fig. 3:**
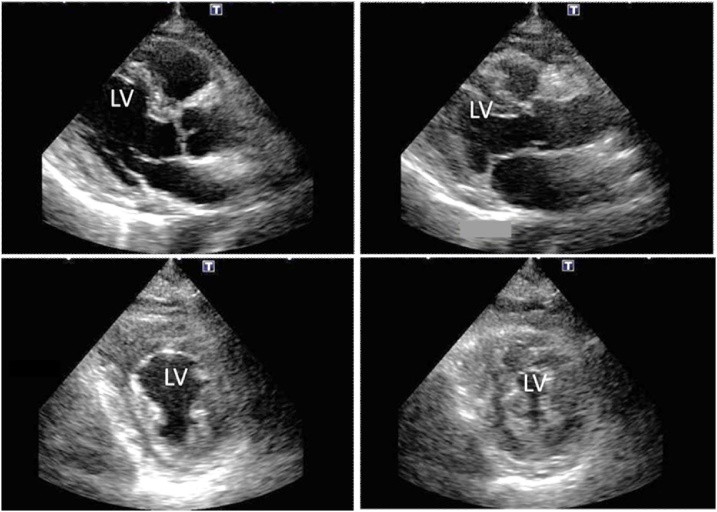
Echocardiogram on POD 44. Parasternal long axis views (top) and short axis views (bottom) of end-systolic frames (right) and end-diastolic frames (left) are shown. Normal left ventricular function with no wall motion abnormalities was found. LV; left ventricular.

The patient showed a good post-operative course and was extubated on post-operative day (POD) 2. She discharged from the ICU on POD 3. On POD 4, she experienced dyspnea and tachycardia. Her blood pressure and oxygenation level had decreased. She had needed re-intubation and ventilation support due to sustained hypotension and hypoxemia. The chest X-ray examination showed expanded cardiothoracic ratio (CTR) and bilateral field opacities in a butterfly distribution, suggesting pulmonary edema due to congestive heart failure (Fig. 1a). Computed tomography revealed diffuse ground-glass appearance in bilateral ling with pleural effusion (Fig. 1b). Echocardiogram showed a severe apical wall motion abnormality with normal basilar wall motion and severely impaired left ventricular function with an EF of 40% (Fig. 2). A 12-lead ECG demonstrated sinus tachycardia and ST-elevation in the all leads and T wave inversion. Blood tests showed that a peak creatine kinase MB (CK-MB) levels were 11.0 IU/L (normal 0–7 IU/L) and troponin I levels were 0.33 ng/mL (normal <0.03 ng/mL). NT- pro BNP, which is an index of heart failure, greatly elevated to 6699 pg/mL (normal <125 pg/mL). Based on these clinical and laboratory findings, especially its characteristic echographic feature, a diagnosis of transient LV-ABS (also referred to as “stress induced cardiomyopathy” or “takotsubo cardiomyopathy”) was made. Because the patient was hemodynamically unstable and had bleeding diathesis, we withheld coronary catheterization although myocardiac infarction could not be excluded completely. We started to administer diuretics, human atrial natriuretic peptide (hANP). The patient’s cardiac and respiratory functions gradually improved. The echocardiography revealed that the EF returned to 62% and she was extubated on POD 9. As improvement of cardiac function, epinephrine was gradually tapered. She was discharged from Intensive Care Unit (ICU) on POD 10. After discharge of ICU, the patient’s subsequent course was unremarkable during the period of hospitalization. NT-pro BNP gradually decreased to 3099 pg/mL, 2655 pg/mL, 2017 pg/mL, and 228 pg/mL on POD 8, 11, 25, and 39. On POD 44, echocardiogram showed normal left ventricular function (the EF 60%) with no wall motion abnormalities (Fig. 3). She was discharged on POD 50 in good condition, with no signs of cardiac insufficiency and good function of liver graft.

## 3 Discussion

A transient LV-ABS (also known as “stress induced cardiomyopathy” or “takotsubo cardiomyopathy”) is a reversible acute dysfunction of left ventricle. This disease typically occurs in postmenopausal women (88%), following an emotionally or physically stressful event [8]. Its clinical features are similar to an acute myocardial infarction. The clinical diagnostic criteria are i) transient akinesis or dyskinesis of the LV apical and mid-ventricular segments with regional wall motion abnormalities extending beyond a single epicardial vascular distribution, ii) absence of obstructive coronary disease or angiographic evidence of acute plaque rupture, iii) new ECG abnormalities such as ST-segment elevation or T-wave inversion, iv) the absence of recent head trauma, intracranial bleeding, pheochromocytoma, obstructive epicardial coronary artery disease, myocarditis, or hypertrophic cardiomyopathy [6]. Since coronary catheterization was not performed due to instability hemodynamics and bleeding diathesis, we could not exclude obstructive coronary disease completely in this case. However, other findings fitted the rest of diagnostic criteria outlined above. Especially, echocardiogram showed characteristic finding of this syndrome and there was no obvious evidence of ischemic heart disease in various other examinations.

The pathophysiology of this disease is unknown, but it is suggested to result from myocardial stunning secondary to high levels of circulating catecholamine and stress-related neuropeptides due to multi-vessel epicardial spasm and myocarditis [1,4,6,9]. The most possible mechanism is that a surge of catecholamines followed a stressful event impairs myocardial perfusion resulting in cardiac myocyte injury.4 In our case, she had undergone stressful liver transplantation which required large volume of transfusion and long operation time.

There is a hypothesis that a decreased level of estrogen which is reported to protect myocardium from the stress of the sympathetic nervous system leads to transient LV-ABS [9,10]. It is also reported that administration of estrogen was able to prevent the onset of transient LV-ABS in postmenopausal women.9 These findings suggest that postmenopausal women such as our case have a high risk of transient LV-ABS because estrogen has decreased. Of note, transient LV-ABS in liver transplantation occurred not only during or immediately after surgery but also on several days after operation (Table 1).

**Table 1:** Reported postoperative transient LV-ABS in liver transplantation.

No.	age	sex	Liver disease	onset post LT	EF(%) at onset	examination for the diagnosis	treatment	outcome	year, ref #
1	65	F	NASH, HCC	4 hours after LT	N/A	EC,UCG, CA	not mentioned	improved	2007 [2]
2	45	F	NASH, LC	witin 2 h afterepurfusion	15	EC,UCG, CA	IABP, vasopressors	improved (deaddue to hemorrage)	2008 [12]
3	60	F	PBC	8 days after LT	20	EC,UCG, CA	metoprolol, aspirin	improved	2009 [14]
4	60	M	LC with hepatitis B	18 days after LT	N/A	EC,UCG, CA	β blocker	improved	2009 [11]
5	62	F	LC with hepatitis C	witin a hour after operation	25	UCG	dobutamine, iloprost, continuous veno nenous haemodialyses	dead	2009 [11]
6	64	M	NASH	15 min after portal reperfusion	35	UCG, CA	temporary transvenous pacemaker, β blocker	improved	2010 [1]
7	33	F	PSC	after liver repurfusion	36	EC,UCG, CA	norepinephrine, dobutamine, β blocker	improved	2012 [15]
8	36	M	LC, Budd-Chiari Syndrome	4 days after LT	25	EC, UCG	norepinephrine, dobutamine	improved (dead due to infection)	2012 [15]
9	51	F	LC, autoimmune hepatitis	2 days after LT	20	EC,UCG, CA	norepinephrine, dobutamine, IABP	improved	2013 [16]
10	52	M	Haemochromatosis	after surgical closure	25	EC,UCG, CA	milrinone, phenylephrine	improved	2014 [17]
11	68	F	LC with hepatitis C, HCC	4 days after LT	40	EC, UCG	diuretics, hANP	improved	our case

NASH; non-alcoholic steatohepatits, HCC; hepatocellular carcinoma, LC; liver cirrhosis, PBC; primary biliary cirrhosis, LT; liver transplantation, EC; electro-cardiogram, UCG; ultrasonic echo-cardiography, CA; coronary antiography, IABP; Intra-aortic balloon pump, hANP; human atrial natriuretic peptide.

When the patient, especially postmenopausal women, complains of chest pain or dyspnea after liver transplantation, the clinician should consider transient LV-ABS although it is relatively rare [2,11,12]. Furthermore, this cardiac complications after surgery, not only at early period after surgery, it is necessary to observe carefully a week before and after surgery.

As for treatment, although optimal management has not been established, it is desirable to avoid or taper catecholamine use if possible and to treat heart failure with diuretics, hAMP, vasodilators, and β-blockers to reduce the pre- and after-load. In a serious case, use of intra-aortic balloon pumping (IABP) can also be considered until the cardiac function improves, and a dynamic blood circulation state should be managed strictly [13]. If the cardiac function is maintained successfully with conservative management, cardiac function will return to a normal level without specific treatment and the prognosis is good in most cases. It is reported that there is no difference between patients with transient LV-ABS and age- and gender-matched general populations in a 4-year survival rate. It is also reported that in-hospital mortality is <2%, and the recurrence rate is generally <10% [4,8]. In the literature, there are 10 cases of the report about transient LV-ABS after liver transplantation except for our case (Table 1). Interestingly, cardiac function had recovered to the normal range in all cases. Therefore, if we are diagnosed with transient LV-ABS, it seems important to manage cardiac function properly and strictly until it improves.

## 4 Conclusion

In conclusion, we report the case of a transient LV-ABS in LDLT recipient. The stress due to invasive surgery such as liver transplantation may be associated with myocardial stunning. It could result in acute heart failure and be a fatal complication after liver transplantation without proper diagnosis and treatment. Transient LV-ABS should be considered as a cause of cardiac dysfunction in recipient of liver transplantation.

## Conflicts of interest

We declare no conflict of interest.

## Sources of funding

This work was supported by the Japan Agency for Medical Research and Development (AMED;JP15fk0210016 h003 and JP16fk0210107).

## Ethical approval

According to our institution guideline, approval to publish this case report was waived by the institution.

## Consent

Written informed consent for publication of this case report and accompanying images was obtained from the patient.

## Author’s contribution

Asuka Tanaka and Takashi Onoe acquired the data, wrote an original draft of the manuscript, revised the draft and approved the final manuscript. Kohei Ishiyama, Kentaro Ide, Hirotaka Tashiro and Hideki Ohdan acquired the data, read and revised the draft and approved the final manuscript.

## Registration of research studies

Not applicable.

## Guarantor

Takashi Onoe and Hideki Ohdan.

## Provenance and peer review

Not commissioned, externally peer-reviewed.
